# Management of a Rare Case of Geminated and Fused Central Incisors in the Same Maxillary Dental Arch

**DOI:** 10.1155/2021/5566827

**Published:** 2021-03-08

**Authors:** Anas Alyahya, Humam Karkoutly, Hussam Milly

**Affiliations:** Department of Conservative Dentistry and Endodontics, Dental Institute, Damascus University, Damascus, Syria

## Abstract

A 27-year-old man presented with developmental disorders in the maxillary incisors and asymmetric smile. Differential diagnosis between geminated and fused maxillary central incisors was conducted using cone-beam computed tomography (CBCT). The complexities of root canal system was handled using suitable shaping instruments and ultrasonic activation tips. Root apexes were sealed with mineral trioxide aggregate plugs. The anterior maxillary gingiva was surgically recontoured, and CAD/CAM Zirconia crowns were placed after the gingival healing phase. The combination of the 3D radiological examination, the clinical screening, and the use of a diagnostic wax-up presented a successful approach to manage this rare clinical case.

## 1. Introduction

Gemination is a malformation of a single tooth during its development to form a completely or partially separated crown with a single root [1]. The union of two or more teeth that normally develops separately is referred as fusion [2]. Gemination exhibits a normal number of teeth, whilst fusion presents a reduced number within the dental arch [3]. The clinical management of teeth with gemination or fusion may present difficulties since the size/shape of the crown deteriorates the aesthetic appearance, and the root canal morphology complicates the endodontic treatment [4].

Visualizing the internal anatomy of roots to obtain sufficient details about their surroundings is critical to achieve successful endodontic treatment [5]. The use of the standard two-dimensional radiographic technologies exhibits a limitation for the localization and characterization of complex root canal systems [6]. The application of cone-beam computed tomography (CBCT**)** has been revealed to be beneficial for endodontic treatment, particularly in understanding the complicated root canal morphology of fused/geminated teeth [7–9]. The flowing report describes multidisciplinary approach to restore the function and the esthetics appearance of both fused and geminated maxillary central incisors with the aid of CBCT.

## 2. Case Presentation

**Figure 1:**
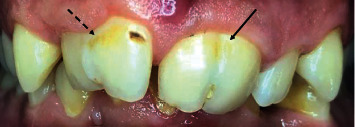
Preoperative image presenting geminated (arrow) and fused maxillary central incisors (dashed arrow).

**Figure 2:**
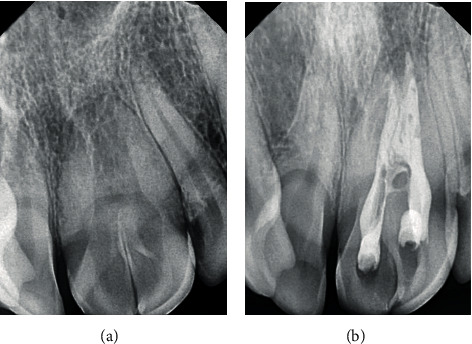
(a) Initial radiograph of tooth #21 before the endodontic intervention. (b) Radiograph of tooth #21 after the endodontic treatment, with the set of the 3 mm apical plugs of MTA.

**Figure 3:**
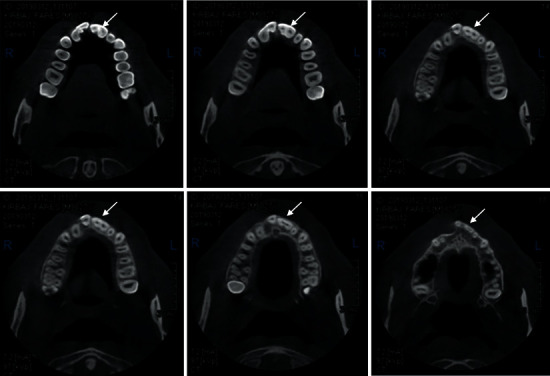
Plane of axial CBCT sections of maxillary showing fusion between #11 and #12 and a gemination in #21. The gemination presents a separated pulp chambers and merging root canals (arrow).

**Figure 4:**
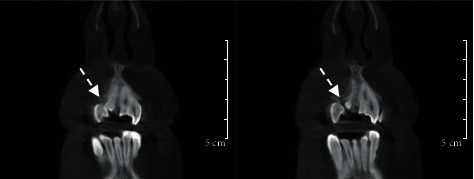
Plane of coronal CBCT sections of maxillary showing the separation level of the fused #11 and #12 (arrow).

A 27-year-old male patient was referred to the Department of Conservative Dentistry and Endodontics with developmental disorders in the maxillary incisors and asymmetric smile. The clinical examination revealed an abnormal morphology of teeth #11 and #21. The absence of #12 and the presence of tooth #22 suggested the probability of fusion between #11 and #12 and a gemination in #21 (Figure 1). All vital signs were found to be normal. The teeth were not sensitive to palpation or percussion. The mobility of the teeth was within the normal limits. Periodontal probing using PCP-UNC 15 probe (Hu-Friedy, Leimen, Germany) showed no periodontal pocketing around the teeth. The two-dimensional radiographic examination (Figure 2) showed a complex pulp structure with no periodontal ligament space around the teeth. CBCT images were obtained using Vatech Pax-Duo3D Pano/CBCT (Vatech™, Seoul, South Korea) unit. CBCT images were reconstructed in three dimensions (3D) using Ez3D-I software (Vatech™, Seoul, South Korea). The CBCT images (Figure 3) revealed separated pulp chambers in the coronal third of #21, then a fusion of both root canals. The teeth #11 and #12 showed a separation under 1 mm of the cemento-enamel junction in the CBCT images (Figure 4).

**Figure 5:**
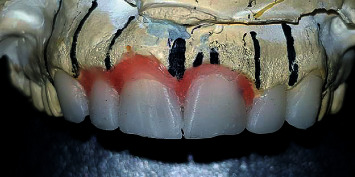
Initial wax-up showing the size/shape of the teeth. A new gingival level was placed.

In this case, the correction of the shape/size of the anterior maxillary teeth would be conducted using fixed prosthesis after crown sectioning and preparation. The potential exposure of pulp chamber of both central incisors indicated the need for root canal treatment, which was conducted before crown preparation and restoration. A diagnostic waxed-up was made (Figure 5), and a mock-up was tried *in vivo*. The treatment plan was discussed thoroughly with the patient, and a written informed consent was obtained.

Anesthesia was administrated, and a rubber dam was applied. Root canals were prepared and shaped using XP-endo Shaper (FKG Dentaire, La Chaux-de-Fonds, Switzerland). Root canals were fully irrigated with 2.5% sodium hypochlorite (NaOCl) and activated using Sonofile K-file tips #25 (Satelec Acteon, Merignac Cedex, France) mounted in a P5 Booster ultrasonic scaler (Satelec Acteon). Apexes were sealed with 3 mm apical MTA (mineral trioxide aggregate) plugs (Dentsply Tulsa Dental, Tulsa, OK, USA). After 24 hours, resin-based sealer (Tgadeal, Technical, and General LTD London) was used with warm gutta-percha (BeeFill 2 in 1, VDW, Munich, Germany) for back-filling (Figure 2). Then, GIC restoration (Fuji IX, GC, Tokyo, Japan) was placed.

**Figure 6:**
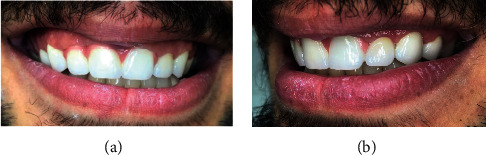
Posttreatment photograph with final result.

A session was set after 2 weeks of the endodontic obturation to recontour the anterior maxillary gingiva. The gingival pocket was 3.5 mm when measured; thus, there was no need for bone crest modification, and the cutting was within 1 mm. The patient returned for the next session after 2 weeks with a completely healed gingiva. The crowns of the anterior maxillary teeth from tooth #13 to tooth #23 were prepared for reshaping and smile correcting according to the cast module analysis. Crown cores were fabricated from zirconia blocks (Vita Zahnfabrik, Bad Säckingen, Germany) using CAD-CAM (computer-aided design and computer-aided manufacturing) system (Roland DWX-52® 5-Axis, California, USA), then gingival color modifiers were added to mimic the surrounding gingiva. The posttreatment photograph with final result is presented in Figure 6.

## 3. Discussion and Conclusion

Dental fusion is the union of two or more dental germs [10]. The fusion maybe a complete or incomplete depending upon stage of teeth development at the time of merging [10]. Such abnormality might be confused with gemination, since gemination is an abnormal development of a tooth separating the crown into two, while sharing a common root [11]. Gemination is the result of a single tooth bud division attempt [12]. Fusion can be differentiated from gemination by the count of teeth within a dental arch and have the number being reduced in fusion whilst remaining normal in gemination [4, 13–15]. In this report, a case of a partially fused central incisor to lateral incisor is presented in the upper right arch. In the upper left arch, however, the number of teeth is normal, and differentiation from gemination is impossible. The differentiation between gemination and fusion may not be critically significant for the clinical treatment [16].

In the dental literature, different clinical approaches are advocated to manage fused/geminated teeth. Those approaches include orthodontic treatment, tooth extraction, reduction of crown size followed by restoration, sectioning of the fused teeth, and no treatment [17].

The developmental anomaly in this clinical case was associated with appearance disapproval and social apprehension, reported by the patient. Therefore, a significant alternation in the teeth shape, size, and proportion was required to achieve a satisfied outcome.

The diagnosis/treatment of fused teeth is intractable. High-resolution 3-dimensional imaging has been demonstrated to be superior to periapical radiography in vitro and in vivo, particularly for canal identification and characterization of internal and external root morphology [18, 19]. In this report, the CBCT images helped in having a 3-dimensional view of the root systems, establishing the fact of having a partial or complete fusion, thus offering the best protocol for endodontic treatment. Wax-up and mock-up revealed the best treatment choices available and enabled the patient to see the expected results before initiating the treatment.

Endodontic treatment was conducted in this case as a significant modification in the incisors' shape was required for esthetic reason. Periodic clinical follow-up is of utmost importance to ascertain the long-term success of the endodontic therapy [20]. The main limitation of the technique used in the management of the present case is that it was not minimally invasive. Esthetic reshaping of anterior teeth required a considerable removal of tooth substance. However, the selection of monolithic zirconia helped in reducing tooth structure loss [21].

Periodic periodontal assessment and treatment are essential for the success of indirect fixed dental prostheses [22]. Different variables affect significantly the prognosis of the treated teeth including oral rinsing solutions, toothbrushing technique, and motivation in self-performed plaque control [23–25].

In conclusion, careful case diagnosis and a precise treatment plan are obligated for the successful management of such rare case. A three-dimensional CBCT introduced valuable information about root canal systems and their surroundings which contributed substantially to the success of the endodontic therapy and case management.

## Data Availability

The data that support the findings of this study are available from the corresponding author upon reasonable request.
